# Triglyceride-cholesterol-body weight index associated with the risk of metabolic dysfunction-associated steatotic liver disease: a population-based cross-sectional study

**DOI:** 10.3389/fnut.2025.1698297

**Published:** 2025-10-30

**Authors:** Li Fan, Yongkang Su, Yue Chen, Ling Xu, Hairong Huang, Chunsheng Lu, Jia Peng, Yingbin Sun, Min Jia

**Affiliations:** ^1^Department of Infection Control of Jiangbei Campus, The First Affiliated Hospital of Army Medical University, Chongqing, China; ^2^Department of Cardiology, The 955th Hospital of the PLA Army, Qamdo, Tibet, China; ^3^Department of Quality Control of Jiangbei Campus, The First Affiliated Hospital of Army Medical University, Chongqing, China; ^4^Outpatient Department of Army Logistics Academy Training Base, The First Affiliated Hospital of Army Medical University, Chongqing, China; ^5^Department of Cardiology, General Hospital of Tibet Military Command, Lhasa, Tibet, China; ^6^Department of Preventive Health Care of Jiangbei Campus, The First Affiliated Hospital of Army Medical University, Chongqing, China

**Keywords:** metabolic dysfunction-associated steatotic liver disease (MASLD), triglyceride-cholesterol-body weight index (TCBI), body mass index (BMI), waist circumference (WC), risk factor

## Abstract

**Background:**

The triglyceride-cholesterol-body weight index (TCBI), a novel parameter integrating serum triglycerides (TG), total cholesterol (TC), and body weight (BW), has not been comprehensively investigated in relation to metabolic dysfunction-associated steatotic liver disease (MASLD). This study aimed to examine the association between TCBI and MASLD.

**Methods:**

This cross-sectional study used data from the NAGALA project in Japan, including 14,242 participants. TCBI was calculated using the formula: TG (mg/dL) × TC (mg/dL) × BW (kg)/1,000. Participants were stratified into quartiles based on natural log-transformed TCBI (TCBI-LN). The association between TCBI and MASLD was assessed using multiple logistic regression, restricted cubic splines (RCS), subgroup analyses, and receiver operating characteristic (ROC) curve analysis.

**Results:**

Among the 14,242 participants, the mean age was 43.53 ± 8.89 years, and 48.03% were female. In the fully adjusted model, each 1-unit increase in TCBI-LN was associated with a 1.69-fold increased risk of MASLD (OR = 2.69, 95% CI: 2.00–3.64, *P* < 0.001). Compared to quartile 1, participants in quartile 4 of TCBI-LN had a 2.91-fold higher risk of MASLD (OR = 2.91, 95% CI: 1.94–4.35, *P* < 0.001). Trend analysis and RCS curve fitting revealed a positive linear and dose-response relationship between TCBI and MASLD risk. Subgroup analysis showed that TCBI was a stronger predictor of MASLD in individuals with abnormal body mass index (BMI) or normal waist circumference (WC). ROC analysis indicated that TCBI had good predictive performance for MASLD among individuals with normal BMI (AUC = 0.810, 95% CI: 0.801–0.822).

**Conclusion:**

Triglyceride-cholesterol-body weight index showed a significant linear positive association with MASLD risk, particularly in individuals with abnormal BMI or normal WC. Moreover, TCBI demonstrated strong predictive accuracy for MASLD among individuals with normal BMI.

## Introduction

Metabolic-associated steatotic liver disease (MASLD) has emerged as a leading chronic liver condition globally, with its prevalence rising markedly in parallel with the epidemics of obesity and type 2 diabetes mellitus (T2DM) (1–5). Current epidemiological data indicate that MASLD affects approximately 38% of adults and 7%–14% of children and adolescents (4, 6), with projections suggesting that prevalence may exceed 55% in adults by 2040 (7). As a multisystem disorder, MASLD pathogenesis is critically driven by insulin resistance (IR) and associated metabolic dysfunction (8–10). It induces progressive hepatic injury ranging from steatosis to cirrhosis, hepatic failure, and hepatocellular carcinoma, and significantly elevates the risk of cardiovascular disease, T2DM, chronic kidney disease, and extrahepatic malignancies, posing a substantial public health burden (8, 11, 12). Consequently, early identification of high-risk individuals and implementation of preventive strategies are imperative to mitigate this burden.

As is well known, obesity, dyslipidemia, and hyperglycemia are established risk factors for MASLD, underscoring its intimate link with metabolic syndrome (13). However, conventional metrics such as isolated lipid parameters or body mass index (BMI) offer limited predictive value: lipid profiles fail to comprehensively represent systemic lipid metabolism, while BMI inadequately characterizes adipose tissue distribution (14). The triglyceride-cholesterol-body weight index (TCBI) is a novel and easily calculable nutritional index that integrates serum triglycerides (TG), total cholesterol (TC), and body weight (BW) (15). The core parameters of TCBI reflect lipid metabolism disorders, energy partitioning, and chronic low-grade inflammation, which may contribute to the “multiple hits” pathogenesis of MASLD. In this process, steatosis and fibrosis progression are collectively driven by lipo-toxicity, IR, and inflammation (16, 17). Furthermore, TCBI has demonstrated prognostic value across various cardiometabolic and neurological disorders, including coronary artery disease (15), acute decompensated heart failure (18), hemodynamically unstable patients (19), stroke (20, 21), and cognitive impairment (22).

Currently, research on the relationship between TCBI and MASLD remains unexplored. Therefore, in this study, we hypothesize that elevated TCBI levels are associated with an increased risk of MASLD, and if so, further evaluate the predictive value of TCBI for MASLD.

## Materials and methods

### Data source and study participants

**FIGURE 1:**
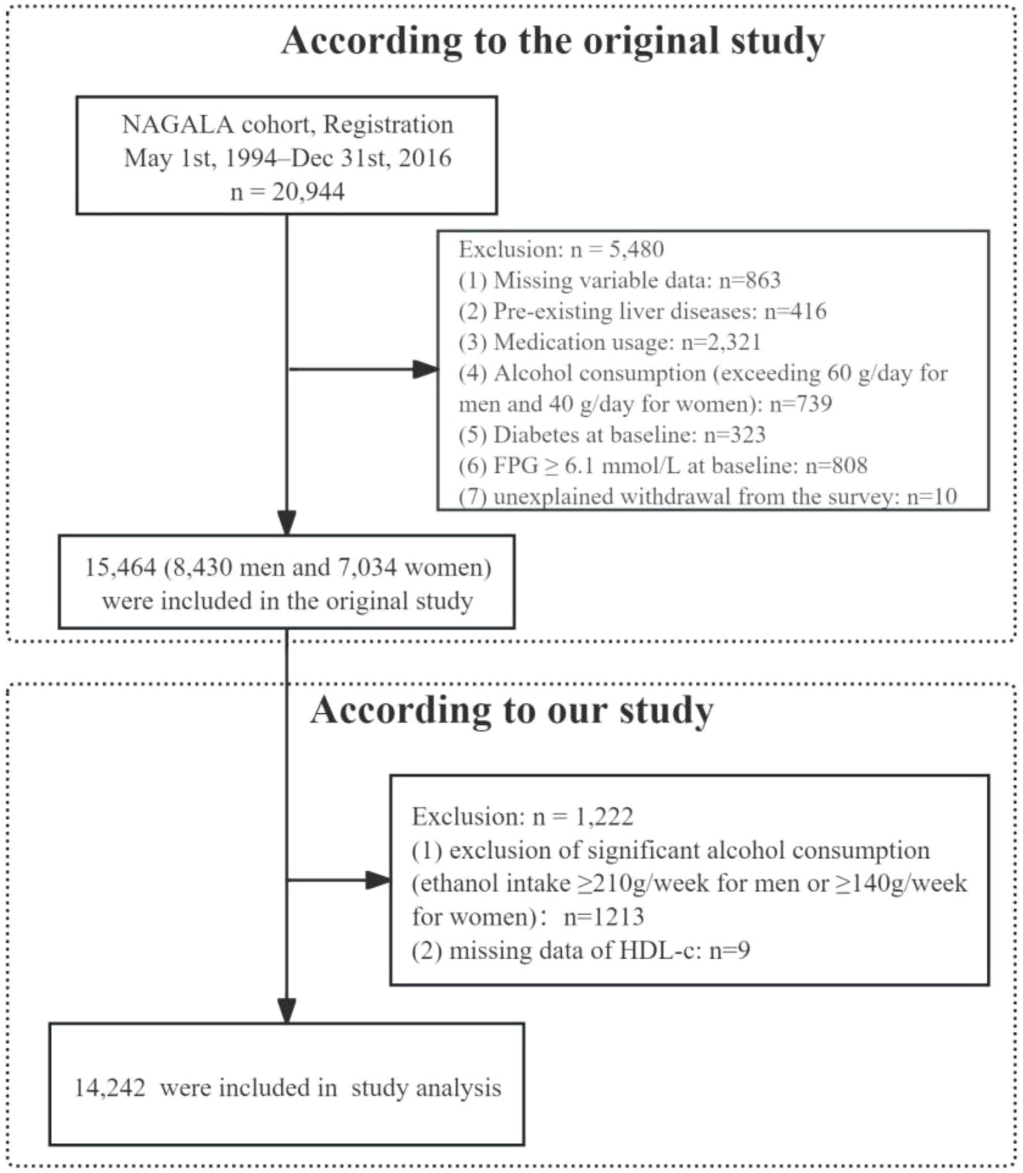
Flowchart of study participants.

This study was conducted as a secondary analysis based on the NAGALA (NAfld in the Gifu Area, Longitudinal Analysis) database, details of which were previously published by Okamura et al. (23). The database data have been deposited in Dryad, an open-access data repository, and are publicly accessible to all researchers^1^. Furthermore, ethical approval and informed consent procedures were described in Okamura et al.’s original study, and our research strictly followed the ethical guidelines outlined in the Declaration of Helsinki. A total of 15,464 participants were initially obtained from the NAGALA database via Dryad. According to our research criteria, we excluded 9 participants who lacked high-density lipoprotein cholesterol (HDL-C) measurements and 1,213 participants whose alcohol consumption was ≥210 g/week for men or ≥140 g/week for women. Consequently, 14,242 participants were included in the final analysis (Figure 1). It is important to note that the specific NAGALA cohort we analyzed, as defined by Okamura et al., explicitly excluded individuals with diabetes at baseline (23). Therefore, our study population inherently consists of participants without pre-existing diabetes.

### Data collection and measurements

Details regarding data collection and measurement methods have been described in a prior study (23). Demographic characteristics of all participants, including age, gender, height, BW, waist circumference (WC), and blood pressure (BP), were collected. BMI was calculated as weight in kilograms divided by height in meters squared. Lifestyle factors, such as smoking status, alcohol consumption, and physical activity, were obtained through a standardized self-reported questionnaire. Alcohol consumption was categorized into three levels: none or minimal (<40 g/week), light (40–139 g/week), and moderate (140–280 g/week) (24). Smoking status was classified into three groups: never, past, and current smoker. Physical activity was defined as a binary variable based on whether individuals engaged in physical activity at least once per week (23). Fasting blood samples were collected from all participants to measure the following biochemical parameters: alanine aminotransferase (ALT), aspartate aminotransferase (AST), gamma-glutamyl transferase (GGT), hemoglobin A1c (HbA1c), fasting plasma glucose (FPG), HDL-C, TG, and TC.

According to established methodologies, TCBI was calculated using the following formula: TCBI = TG (mg/dL) × TC (mg/dL) × BW (kg)/1,000 (15).

### MASLD diagnosis

Metabolic dysfunction-associated steatotic liver disease was defined based on the multi-society Delphi consensus criteria developed by the American Association for the Study of Liver Diseases (25). Hepatic steatosis was diagnosed using abdominal ultrasonography conducted by trained technicians, with independent confirmation provided by two gastroenterologists (26). The diagnosis of MASLD requires meeting all of the following criteria: (1) absence of significant alcohol consumption (ethanol intake ≥ 210 g/week for men or ≥140 g/week for women); (2) presence of hepatic steatosis; and (3) presence of at least one of the following five cardiometabolic risk factors: TG ≥ 150 mg/dL or use of lipid-lowering therapy; HDL-C ≤ 40 mg/dL for men and ≤50 mg/dL for women or use of lipid-lowering therapy; FPG ≥ 100 mg/dL or HbA1c ≥ 5.7%, or history of T2DM; BMI ≥ 23.0 kg/m^2^ (using Asian-specific cutoffs) or WC > 94.0 cm for men and >80.0 cm for women; and BP ≥ 130/85 mmHg or use of antihypertensive therapy.

### Statistical analysis

**FIGURE 2:**
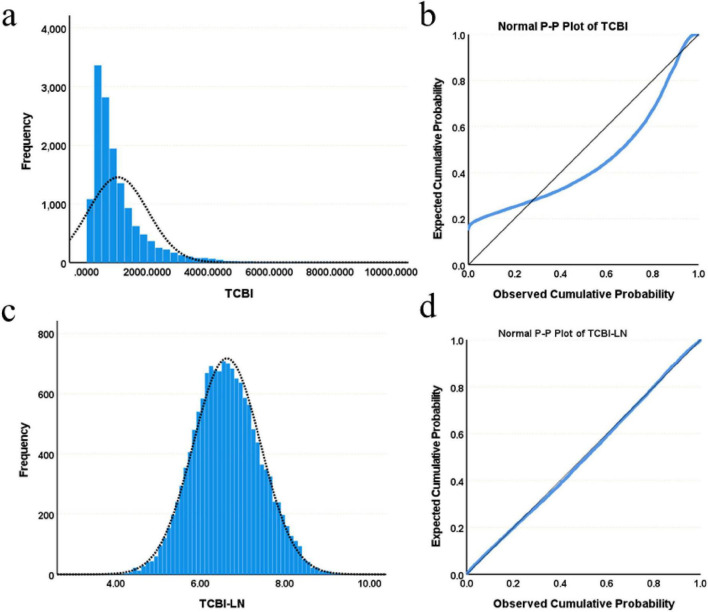
Distribution characteristics of TCBI and TCBI-LN. **(a)** Histogram of TCBI. **(b)** Normal P-P plot of TCBI. **(c)** Histogram of TCBI-LN. **(d)** Normal P-P plot of TCBI-LN.

Baseline characteristics were summarized as follows: continuous variables with normal distribution were presented as mean ± standard deviation (SD), skewed variables as median (interquartile range), and categorical variables as frequency (percentage). Normality was assessed using histograms, probability-probability (P-P) plots, and the Kolmogorov-Smirnov test. Due to its skewed distribution, the TCBI variable was natural log-transformed to approximate a normal distribution, and the resulting transformed variable, referred to as TCBI-LN, was utilized in subsequent statistical analyses (Figure 2). Participants were categorized into quartiles based on TCBI-LN cutoffs to enable systematic comparison of baseline characteristics across these strata [TCBI-LN quartiles: Quartile 1 (≤6.08), Quartile 2 (6.09–6.60), Quartile 3 (6.61–7.15), Quartile 4 (>7.15)]. Continuous variables were compared using one-way ANOVA or the Kruskal-Wallis H test, depending on the homogeneity of variance and normality; categorical variables were analyzed using the chi-square test or Fisher’s exact test. To assess the correlation between baseline parameters and TCBI, Pearson correlation coefficients were calculated.

Multivariate logistic regression analyses were conducted to evaluate the association between TCBI and the risk of MASLD, with results expressed as odds ratios (OR) per unit increase and their corresponding 95% confidence intervals (CI). Both crude and adjusted models were constructed to account for potential confounding variables: Model 1 was crude; Model 2 adjusted for sex and age; Model 3 further adjusted for exercise habits, alcohol consumption, smoking status, BMI, and WC; Model 4 additionally adjusted for ALT, AST, GGT, HDL-C, HbA1c, FPG, systolic blood pressure (SBP), and diastolic blood pressure (DBP). A trend test was performed to evaluate dose-response relationships across quartiles of TCBI-LN. In addition, restricted cubic splines (RCS) were used to visualize the potential non-linear relationship between TCBI and MASLD prevalence. Stratified logistic regression analyses were carried out according to sex, WC, age, BMI (18.5–23.0 kg/m^2^ vs. <18.5 or ≥23.0 kg/m^2^), physical activity, alcohol consumption, smoking status, and hypertension status to explore the association between TCBI and MASLD within different subgroups. Finally, receiver operating characteristic (ROC) curves and the area under the curve (AUC) were generated to evaluate the predictive performance of TCBI for MASLD risk. A *post hoc* power analysis was performed using G*Power 3.1 software, which confirmed that the present sample size provided >99% power to detect the observed association (OR = 2.69 for TCBI-LN) at a significance level of α = 0.05, indicating sufficient statistical power.

All statistical analyses and graphical presentations were performed using SPSS version 27.0 (IBM Corporation, USA) and R version 4.4.2. A two-sided *p*-value < 0.05 was considered statistically significant.

## Results

### Baseline characteristics

**FIGURE 3:**
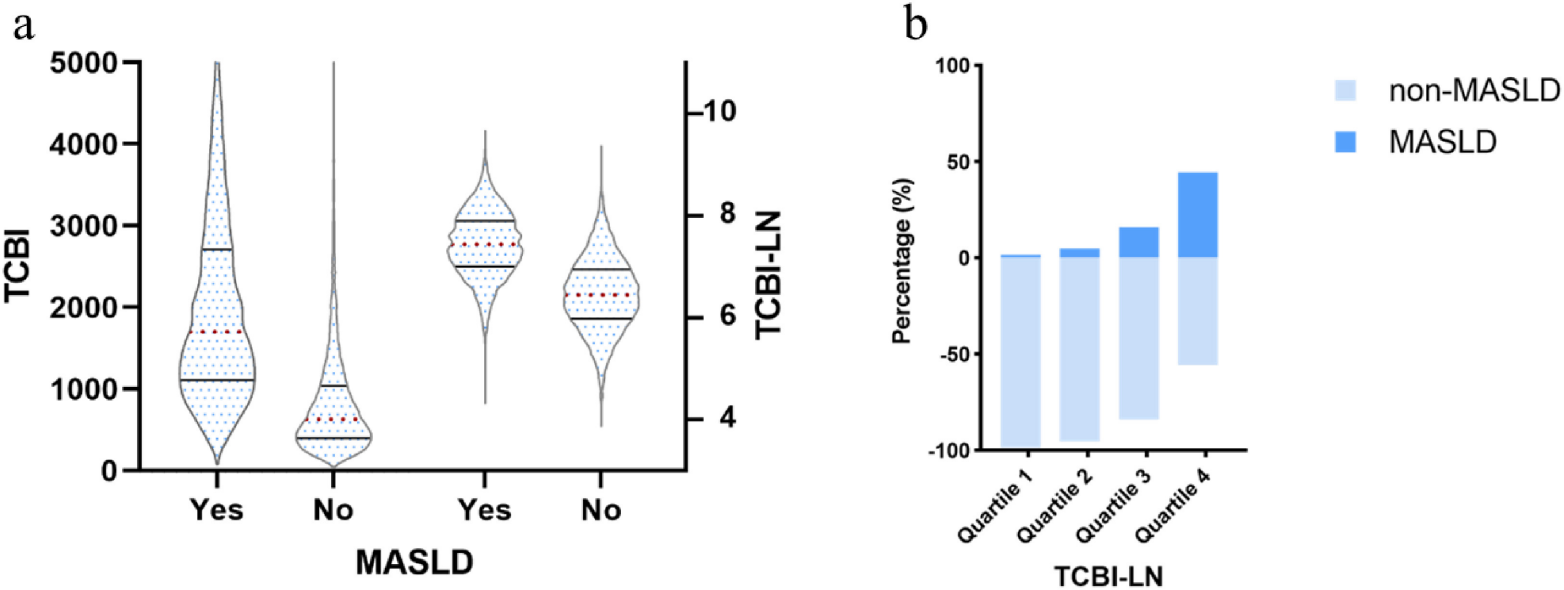
**(a)** Distribution of TCBI and TCBI-LN according to MASLD status; **(b)** Prevalence of MASLD across TCBI-LN quartiles by diagnostic category.

As presented in Table 1, a total of 14,242 participants, with a mean age of 43.53 ± 8.89 years and 48.03% female, were categorized into four groups based on TCBI-LN, the natural logarithm of TCBI. Comparative analysis across groups demonstrated statistically significant differences in all measured variables (all *P* < 0.05). With ascending TCBI-LN quartiles, there were progressive increases in age, BMI, BW, WC, ALT, AST, GGT, TC, TG, HbA1c, FPG, SBP, and DBP, while HDL-C levels showed a consistent decline. Additionally, the proportions of women, individuals engaging in regular exercise, non-drinkers, and never-smokers were lower in the higher TCBI-LN quartiles. Notably, participants with MASLD exhibited significantly higher TCBI-LN values compared to those without MASLD; Furthermore, the prevalence of MASLD increased progressively from Quartile 1 to Quartile 4 (Figure 3).

**TABLE 1:** Baseline characteristics of participants across TCBI-LN quartiles.

Variables	Overall	TCBI-LN quartiles				
		Quartile 1	Quartile 2	Quartile 3	Quartile 4	*P*
*N*	14242	3561	3560	3561	3560	
Age (year)	43.53 ± 8.89	39.98 ± 8.17	43.46 ± 8.77	45.33 ± 8.97	45.37 ± 8.55	<0.001
Sex (female)	6840 (48.03%)	2840 (79.75%)	2053 (57.67%)	1326 (37.25%)	621 (17.44%)	<0.001
BMI (kg/m^2^)	22.06 ± 3.14	19.92 ± 2.14	21.14 ± 2.42	22.58 ± 2.65	24.62 ± 3.10	<0.001
BW (kg)	60.26 ± 11.61	51.83 ± 7.65	56.77 ± 9.01	62.23 ± 9.63	70.20 ± 11.00	<0.001
WC (cm)	76.18 ± 9.10	69.50 ± 6.46	73.29 ± 7.29	77.91 ± 7.51	84.03 ± 7.89	<0.001
ALT (IU/L)	16.00 (12.00, 23.00)	13.00 (11.00, 17.00)	15.00 (12.00,19.00)	17.00 (14.00, 23.00)	23.00 (17.00,33.00)	<0.001
AST (IU/L)	17.00 (14.00, 21.00)	16.00 (13.00, 19.00)	16.00 (14.00,20.00)	17.00 (14.00, 21.00)	19.00 (16.00,24.00)	<0.001
GGT (IU/L)	15.00 (11.00, 21.00)	12.00 (10.00, 14.00)	13.00 (10.00,17.00)	16.00 (12.00, 22.00)	22.00 (16.00,32.00)	<0.001
HDL-C (mg/dL)	56.46 ± 15.48	64.37 ± 14.56	61.18 ± 14.91	54.91 ± 14.03	45.37 ± 10.83	<0.001
TC (mg/dL)	198.14 ± 33.57	174.83 ± 26.24	192.44 ± 27.96	204.60 ± 29.18	220.70 ± 32.56	<0.001
TG (mg/dL)	64.00 (43.00, 97.00)	34.00 (27.00, 40.00)	53.00 (46.75,61.00)	77.00 (67.00, 89.00)	132.00 (108.00,169.00)	<0.001
HbA1c (%)	5.18 ± 0.32	5.12 ± 0.30	5.15 ± 0.31	5.20 ± 0.32	5.24 ± 0.34	<0.001
FPG (mg/dL)	92.73 ± 7.42	88.87 ± 6.74	91.49 ± 7.15	94.04 ± 6.86	96.51 ± 6.63	<0.001
SBP (mmHg)	113.93 ± 14.82	106.73 ± 12.50	111.16 ± 13.42	116.19 ± 14.22	121.64 ± 14.70	<0.001
DBP (mmHg)	71.12 ± 10.38	65.81 ± 8.75	69.14 ± 9.36	72.77 ± 9.93	76.76 ± 10.09	<0.001
Exercise						0.003
No	11774 (82.67%)	2961 (83.15%)	2876 (80.79%)	2943 (82.67%)	2994 (84.08%)	
Yes	2468 (17.33%)	600 (16.85%)	684 (19.21%)	617 (17.33%)	567 (15.92%)	
Drinking						<0.001
None	11802 (82.87%)	3231 (90.73%)	2985 (83.85%)	2865 (80.48%)	2721 (76.41%)	
Light	1754 (12.32%)	279 (7.83%)	441 (12.39%)	491 (13.79%)	543 (15.25%)	
Moderate	686 (4.82%)	51 (1.43%)	134 (3.76%)	204 (5.73%)	297 (8.34%)	
Smoking						<0.001
Never	8742 (61.38%)	2852 (80.09%)	2450 (68.82%)	1948 (54.72%)	1492 (41.90%)	
Past	2556 (17.95%)	361 (10.14%)	535 (15.03%)	760 (21.35%)	900 (25.27%)	
Current	2944 (20.67%)	348 (9.77%)	575 (16.15%)	852 (23.93%)	1169 (32.83%)	
MASLD	2333 (16.38%)	45 (1.26%)	160 (4.49%)	558 (15.67%)	1570 (44.09%)	<0.001

### Correlation analysis

**FIGURE 4:**
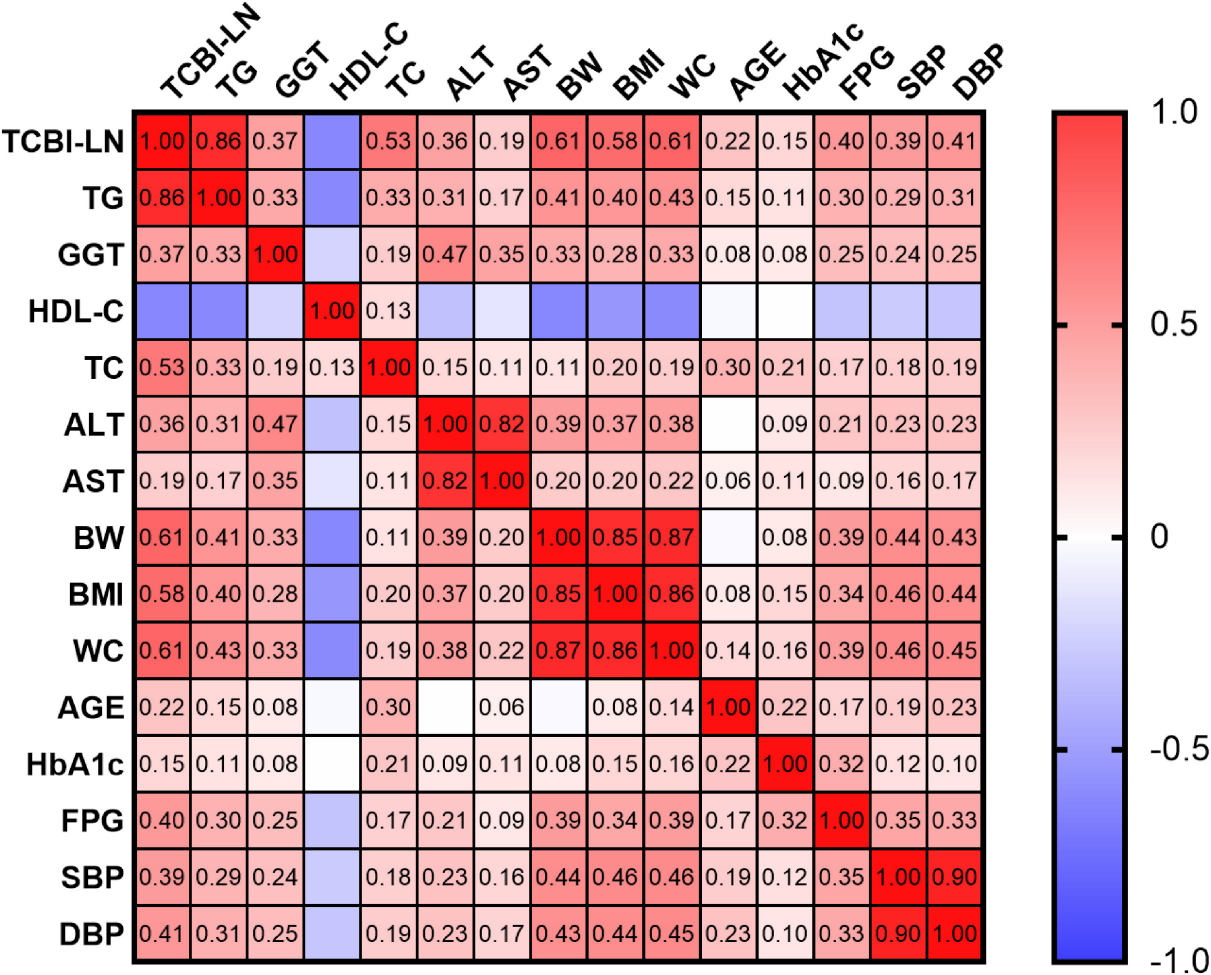
Correlation heatmap illustrating the relationships between TCBI-LN and baseline characteristics.

Pearson correlation analysis was conducted to assess the associations between TCBI-LN and various metabolic and anthropometric parameters. TCBI-LN showed statistically significant correlations with all analyzed variables (all *P* < 0.001). TG exhibited the strongest positive correlation with TCBI-LN compared to other parameters (*r* = 0.86 for TG; *r* = 0.61 for BW; *r* = 0.61 for WC; *r* = 0.58 for BMI; *r* = 0.53 for TC; *r* = 0.41 for DBP; *r* = 0.40 for FPG; *r* = 0.39 for SBP; *r* = 0.37 for GGT; *r* = 0.36 for ALT; *r* = 0.22 for age; *r* = 0.19 for AST; *r* = 0.15 for HbA1c), with the exception of HDL-C (*r* = −0.47) (Supplementary Table 1). The correlation heatmap further illustrated a consistent pattern of metabolic disturbances and highlighted strong interrelationships among key components of metabolic syndrome and hepatic function markers associated with elevated TCBI-LN levels (Figure 4).

### Association between TCBI and MASLD

**FIGURE 5:**
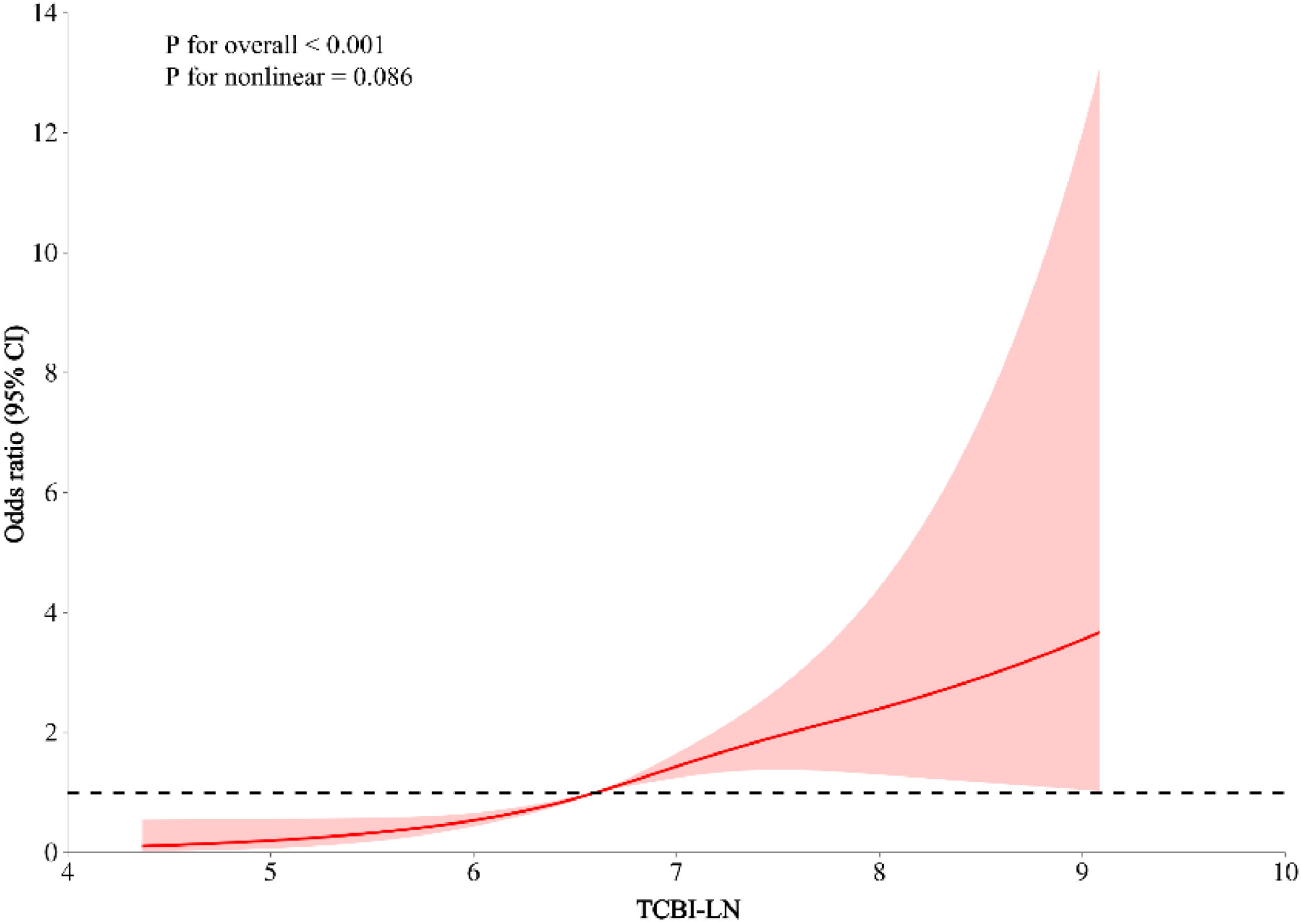
Linear association between TCBI and MASLD after adjusting for sex, exercise, drinking, smoking, age, BMI, WC, ALT, AST, GGT, HDL-C, TC, TG, HbA1c, FPG, SBP, and DBP.

Multivariable logistic regression models were employed to investigate the association between TCBI and MASLD (Table 2). In the crude model (Model 1), each 1-unit increase in TCBI-LN was associated with a 6.36-fold higher likelihood of MASLD prevalence (OR = 7.36, 95% CI: 6.77–8.01, *P* < 0.001). This positive association remained statistically significant across progressively adjusted models: Model 2 (adjusted for sex and age), Model 3 (Model 2 plus exercise, alcohol consumption, smoking status, BMI, and WC), and Model 4 (Model 3 plus ALT, AST, GGT, HDL-C, TC, TG, HbA1c, FPG, SBP, and DBP). After full adjustment in Model 4, each 1-unit increase in TCBI-LN was associated with a 1.69-fold elevated risk of MASLD (OR = 2.69, 95% CI: 2.00–3.64, *P* < 0.001). When TCBI-LN was analyzed as a categorical variable by quartiles, using the lowest quartile (Quartile 1) as the reference group, the Quartile 2, Quartile 3, and Quartile 4 groups exhibited significantly higher MASLD prevalence by 0.52-fold (OR = 1.52, 95% CI: 1.05–2.20, *P* < 0.05), 1.35-fold (OR = 2.35, 95% CI: 1.64–3.37, *P* < 0.001), and 1.91-fold (OR = 2.91, 95% CI: 1.94–4.35, *P* < 0.001) (*P* for trend < 0.001) respectively. RCS curve fitting analysis was conducted to explore the dose-response relationship between TCBI and the prevalence of MASLD, revealing a linear increase in MASLD risk with increasing TCBI levels (overall *P* < 0.001; *P* for non-linear = 0.086) (Figure 5).

**TABLE 2:** Multivariate logistic regression analyses for the association between TCBI and MASLD.

Variables	Model 1	Model 2	Model 3	Model 4
	OR (95% CI)	OR (95% CI)	OR (95% CI)	OR (95% CI)
TCBI-LN	7.36 (6.77, 8.01)***	6.49 (5.94, 7.10)***	3.04 (2.75, 3.37)***	2.69 (2.00, 3.64)***
**TCBI-LN quartiles**				
Quartile 1	1.00 (reference)	1.00 (reference)	1.00 (reference)	1.00 (reference)
Quartile 2	3.68 (2.63, 5.14)***	3.19 (2.28, 4.47)***	1.72 (1.22, 2.45)**	1.52 (1.05, 2.20)*
Quartile 3	14.52 (10.67, 19.75)***	11.28 (8.24, 15.45)***	3.58 (2.59, 4.97)***	2.35 (1.64, 3.37)***
Quartile 4	61.64 (45.60, 83.32)***	43.48 (31.85, 59.35)***	7.90 (5.71, 10.94)***	2.91 (1.94, 4.35)***
*P* for trend	<0.001	<0.001	<0.001	<0.001

Model 1: crude model. Model 2: adjust: sex, age. Model 3: adjust: sex, exercise, drinking, smoking, age, BMI, WC. Model 4: adjust: sex, exercise, drinking, smoking, age, BMI, WC, ALT, AST, GGT, HDL-C, TC, TG, HbA1c, FPG, SBP, DBP. OR, odds ratio; CI, confidence interval; TCBI-LN, log-transformed TCBI.

*P < 0.05;

**P < 0.01;

***P < 0.001.

### Subgroup analyses by adjusted potential effect confounders

**FIGURE 6:**
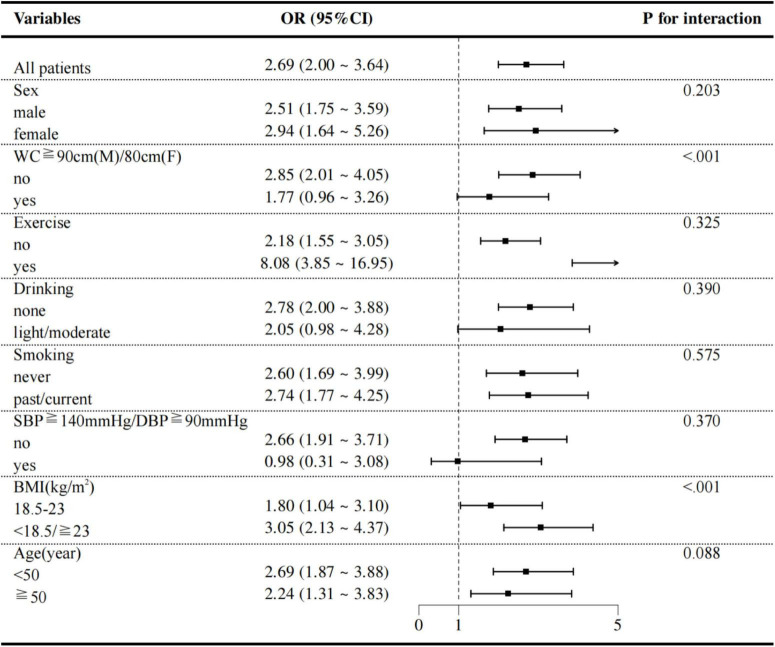
Subgroup analyses of potential effect modifiers on the association between TCBI and MASLD.

The robustness of the association between TCBI and MASLD was further examined through several subgroup and interaction analyses. As shown in Figure 6, the relationship between TCBI-LN and MASLD remained consistent across subgroups defined by sex (male or female), exercise (no or yes), drinking status (none or light/moderate), smoking status (never or past/current), presence of SBP ≥ 140 mmHg or DBP ≥ 90 mmHg (no or yes), and age ≥ 50 years (no or yes). In contrast, significant interaction effects were observed for both BMI and WC (*P* for interaction < 0.01). Specifically, the positive association was more pronounced among individuals with normal WC (OR = 2.85, 95% CI: 2.01–4.05) and those who were underweight or overweight/obese (OR = 3.05, 95% CI: 2.13–4.37).

### Evaluate the accuracy of TCBI in identifying MASLD

**FIGURE 7:**
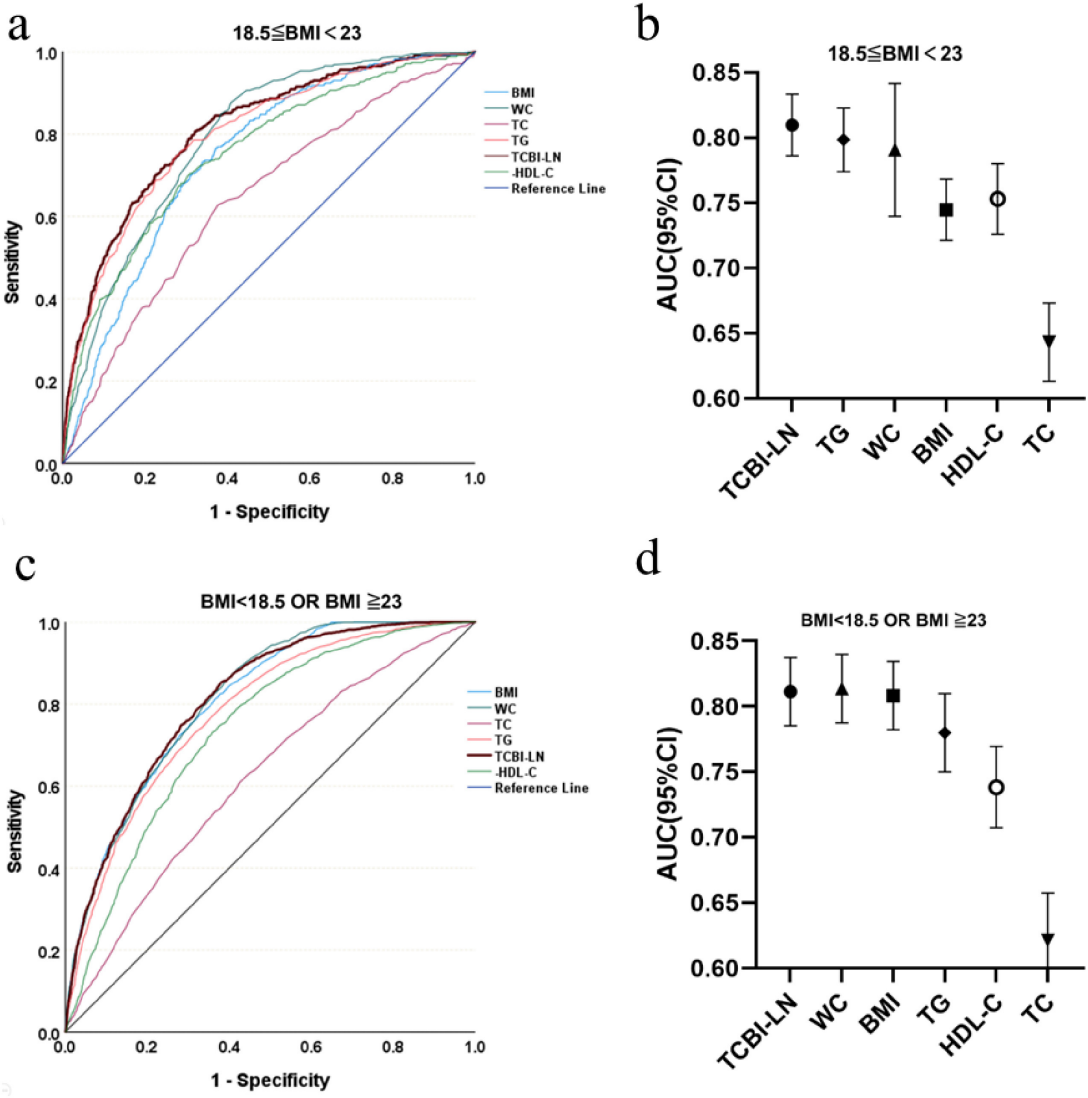
Diagnostic accuracy of TCBI for MASLD risk stratification across BMI categories. **(a)** ROC curves for BMI 18.5–23. **(b)** AUC values for BMI 18.5–23. **(c)** ROC curves for BMI < 18.5 or ≥ 23. **(d)** AUC values for BMI < 18.5 or ≥ 23.

Receiver operating characteristic analysis was used to evaluate the predictive value of TCBI-LN in MASLD risk stratification according to BMI status (Figure 7 and Supplementary Table 2). Notably, in individuals with normal BMI, the predictive performance of TCBI-LN (AUC = 0.810, 95% CI: 0.786–0.833) was superior to that of individual conventional metrics, including TG (AUC = 0.798, 95% CI: 0.774–0.823), WC (AUC = 0.791, 95% CI: 0.770–0.811), HDL-C (AUC = 0.753, 95% CI: 0.726–0.780), BMI (AUC = 0.745, 95% CI: 0.721–0.768), and TC (AUC = 0.643, 95% CI: 0.613–0.673). In contrast, among individuals who were underweight, overweight, or obese, the predictive performance of TCBI-LN (AUC = 0.811, 95% CI: 0.801–0.822) was comparable to that of WC (AUC = 0.814, 95% CI: 0.803–0.824).

## Discussion

This study, based on the East Asian population dwelling in the Gifu area of Japan, is the first to reveal the independent and positive association between TCBI and the risk of MASLD. Our data show that the association between TCBI and MASLD is nearly robust whether TCBI is treated as a continuous or categorical variable, regardless of whether in the crude or the fully adjusted model. In addition, the trend analysis and RCS curves indicate that the relationship between TCBI and MASLD is not only potentially linear but also dose-response. It is to be note that a higher TCBI indicates a higher risk of MASLD in individuals with abnormal BMI while shows better predictive value in those with normal BMI, suggesting that TCBI may be particularly applicable to identify one latent MASLD individual with the normal BMI but abnormal metabolism condition.

Triglyceride-cholesterol-body weight index, one composite indicator integrating TG, TC and BW, involves lipid metabolism disorder and energy surplus which are the core pathogenic factors of MASLD (27). Correlation analysis suggests that TCBI has the strongest correlation with TG (*r* = 0.86, *P* < 0.001), followed by BW and WC (*r* = 0.61, *P* < 0.001), and is negatively correlated to HDL-C (*r* = −0.47, *P* < 0.001). This correlation pattern accords with the pathophysiologic core of MASLD that elevated TG indicating the dysregulation of synthesis and transport of lipid in liver, abnormal TC revealing the disorder of cholesterol metabolism, and elevated BW directly related to excessive fat accumulation (28). Besides, the multiplication form of TCBI in part amplifies the synergistic effect between lipid and obesity, more able to reflect the cumulative load of multiple-hits hypothesis compared to the single factor.

Our analysis reveals a robust, dose-response association between TCBI and MASLD that was consistent across various model specifications and extensive covariate adjustments. This robust statistical association is mechanistically grounded in the TCBI formula itself, which integrates triglycerides (reflecting hepatic lipo-toxicity), total cholesterol (indicating cholesterol metabolic homeostasis), and body weight (a proxy for energy surplus and adiposity). Collectively, these components point directly to the core pathophysiological pathways of MASLD: lipo-toxicity, IR, and the associated chronic inflammatory state (29, 30). While our dataset lacks direct inflammatory biomarkers, the metabolic disturbances quantified by TCBI are well-established drivers of a pro-inflammatory state. Therefore, TCBI appears to serve as an integrative marker of the synergistic metabolic insults that contributes to the “multiple hits” hypothesis of MASLD.

Several previous studies had investigated the predictive value of TCBI in other organ systems and found that TCBI was negatively associated with stroke-associated adverse outcomes (21, 31), cognitive impairment (22), hypertension-associated stroke (20), and prognosis of cardiovascular diseases (32–34). These findings indicate that nutritional energy reserve faces great challenge when the body suffers external (such as trauma) or internal (such as aging) stressors. That is to say, nutrition state reflected by high TCBI might to predict metabolic dysregulation in normal homeostasis; in contrast, it might to indicate anti-stress capacity of the body in dysregulated homeostasis (35).

In addition, subgroup analyses revealed variations in the strength of the association between TCBI and MASLD across BMI and WC categories. The association was significantly stronger among individuals with abnormal BMI (defined as <18.5 or ≥23.0 kg/m^2^), where each unit increase in TCBI was associated with a substantially higher risk of MASLD (OR = 3.05, 95% CI: 2.13–4.37), compared to those with normal BMI (OR = 1.80, 95% CI: 1.04–3.10). A similar pattern was observed in individuals with normal WC (OR = 2.85, 95% CI: 2.01–4.05). In contrast, the association was attenuated and did not reach statistical significance in the abdominal obesity subgroup (OR = 1.77, 95% CI: 0.96–3.26). The distinct patterns of association between TCBI and MASLD across BMI and WC subgroups can be interpreted through the interplay of lipid metabolism, IR, and inflammation. In individuals with abnormal BMI, the stronger association may stem from a synergistic amplification between the lipid abnormalities captured by TCBI and the more severe IR typically present in this group, which collectively accelerate hepatic steatosis and injury (36, 37). Conversely, in participants with abdominal obesity, the association was attenuated. This may be because in such individuals, MASLD risk is predominantly driven by visceral adipose tissue-derived inflammation, which might partially overshadow the contribution of circulating lipids as reflected by TCBI (38, 39). The robust association observed in those with normal WC further affirms TCBI’s ability to detect early metabolic dysregulation even in the absence of overt central obesity, highlighting its clinical relevance for identifying metabolically unhealthy individuals with normal adiposity distribution.

Furthermore, our ROC analysis demonstrated that TCBI had better predictive power for MASLD than traditional single parameters like BMI, WC, TG, or HDL-C among individuals with normal BMI. Collectively, our findings position TCBI as a unique tool for risk stratification. Its particular value lies in identifying high-risk individuals with normal BMI but disturbed lipid metabolism, consequently demonstrating the power of a composite index to capture multifaceted metabolic dysfunction that single metrics may miss. From a clinical perspective, the computational simplicity of TCBI, derived from routine laboratory and anthropometric data, positions it as a potential first-line screening tool for MASLD risk stratification, particularly in primary care. Its clinical utility may be most pronounced in identifying individuals with normal BMI yet elevated metabolic risk. In such cases, an elevated TCBI could serve as an indicator for referral to more definitive, albeit costlier, diagnostic procedures such as liver ultrasonography or vibration-controlled transient elastography. This approach could enhance the detection of the lean MASLD phenotype, facilitating earlier intervention.

### Study strengths and limitations

The primary strength of this study lies in being the first to investigate the association between TCBI and MASLD, uncovering a novel positive linear relationship. Comprehensive statistical adjustments were performed to account for multiple confounding factors, including anthropometric, lifestyle, and biochemical variables. Furthermore, the large sample size enhances the robustness and reliability of the findings.

However, several limitations should be acknowledged when interpreting these results. First and foremost, the cross-sectional design of this study inherently precludes causal inference. Although we observed a robust association between TCBI and MASLD after extensive adjustment for confounders, this finding should be interpreted as identifying a significant risk indicator rather than proving causation. In future, prospective longitudinal studies are essential to verify the temporal sequence and any potential causal link. Furthermore, the design cannot rule out the possibility of reverse causality, whereby pre-existing MASLD could exacerbate dyslipidemia and metabolic dysregulation, thereby influencing the components of TCBI. This potential bidirectional relationship also warrants clarification in future prospective longitudinal studies. Second, the external validity of our findings requires further investigation. Our results were derived from a single, ethnically homogeneous Japanese cohort and lack validation in an independent external cohort. This may limit the generalizability of our findings to other populations. Future studies involving multi-ethnic and geographically diverse cohorts are necessary to validate the association and predictive performance of TCBI for MASLD. Third, our findings are derived from a cohort that excluded individuals with diabetes at baseline. Thus, our study population does not represent the full spectrum of MASLD, it may result in an underestimation of the overall MASLD risk in the general population and limits the generalizability of our results to populations that include diabetic individuals. Fourth, although we adjusted for major known confounding factors, residual confounding due to unmeasured variables–such as dietary patterns, gut microbiota composition, and genetic background–cannot be ruled out and may affect the interpretation of our findings. Fifth, we acknowledge a potential for circularity because TG and BW are used both to calculate the TCBI and as components of the MASLD diagnostic criteria. This overlap may inflate the strength of the observed association. Therefore, TCBI is best viewed as a practical integrator of these core metabolic risk factors rather than as a completely independent predictor. Finally, TCBI was assessed only at baseline, which limits our ability to evaluate its dynamic changes over time in relation to MASLD development.

## Conclusion

In conclusion, TCBI demonstrates a significant and linear positive association with MASLD risk. As an easily accessible composite index, it shows strong potential for integration into clinical practice to identify high-risk individuals, especially those with normal BMI but underlying metabolic dysfunction, thereby facilitating targeted prevention and early intervention strategies.

## Data Availability

The datasets presented in this study can be found in online repositories. The names of the repository/repositories and accession number(s) can be found below: The raw data can be accessed at: https://doi.org/10.5061/dryad.8q0p192.

## Footnotes

1
https://doi.org/10.5061/dryad.8q0p192
