# Supplementary material for: Triglyceride-cholesterol-body weight index associated with the risk of metabolic dysfunction-associated steatotic liver disease: a population-based cross-sectional study
Source: Front Nutr. 2025 Oct 30;12:1698297. doi: 10.3389/fnut.2025.1698297 (PMC12611659; doi:10.3389/fnut.2025.1698297)
Supplement: Supplementary file 1 [file Table_1.docx]

**Supplementary Table S1** The correlation between TCBI-LN and baseline various parameters

| **Variables** | **Factor** | **Correlation** | ***P*** |
| --- | --- | --- | --- |
| TG | TCBI-LN | 0.86 | <0.001 |
| GGT |  | 0.37 | <0.001 |
| HDL-C |  | -0.47 | <0.001 |
| TC |  | 0.53 | <0.001 |
| ALT |  | 0.36 | <0.001 |
| AST |  | 0.19 | <0.001 |
| BW |  | 0.61 | <0.001 |
| BMI |  | 0.58 | <0.001 |
| WC |  | 0.61 | <0.001 |
| Age |  | 0.22 | <0.001 |
| HbA1c |  | 0.15 | <0.001 |
| FPG |  | 0.40 | <0.001 |
| SBP |  | 0.39 | <0.001 |
| DBP |  | 0.41 | <0.001 |

**Supplementary Table S2** The AUC of TCBI for predicting MASLD across different BMI categories

| **Variables** | **Normal BMI** | | | **Abnormal BMI** | | |
| --- | --- | --- | --- | --- | --- | --- |
|  | **AUC** | **95% CI low** | **95% CI up** | **AUC** | **95% CI low** | **95% CI up** |
| BMI | 0.808 | 0.798 | 0.819 | 0.745 | 0.721 | 0.768 |
| WC | 0.814 | 0.803 | 0.824 | 0.791 | 0.770 | 0.811 |
| TC | 0.621 | 0.607 | 0.636 | 0.643 | 0.613 | 0.673 |
| TG | 0.780 | 0.768 | 0.792 | 0.798 | 0.774 | 0.823 |
| TCBI-LN | 0.811 | 0.801 | 0.822 | 0.810 | 0.786 | 0.833 |
| HDL-C | 0.738 | 0.726 | 0.751 | 0.753 | 0.726 | 0.780 |
